# New Interpretation of Glass Formation in Isomeric Substances: Shifting from Melting‐Point to Melting‐Entropy

**DOI:** 10.1002/advs.202206389

**Published:** 2023-02-15

**Authors:** Baokang Ren, Zijing Li, Yanhui Zhang, Shidong Feng, Li‐Min Wang

**Affiliations:** ^1^ State Key Lab of Metastable Materials Science and Technology School of Materials Science and Engineering Yanshan University Qinhuangdao Hebei 066004 China; ^2^ Key Lab for Microstructural Material Physics of Hebei Province School of Science Yanshan University Qinhuangdao Hebei 066004 China

**Keywords:** glass‐forming ability, isomer, melting entropy, melting point

## Abstract

Revealing the critical thermodynamic parameters determining the glass formation of substances is of great significance for understanding the glass transition and guiding the composition design of glass‐forming materials. Nevertheless, the direct access to glass‐forming ability (GFA) by thermodynamics for various substances remains to be substantiated. The strategy to seek the fundamental properties of glass formation is explored several decades ago, as pioneered by Angell, arguing that the GFA in isomeric xylenes depends on the low lattice energy manifested by the low melting point. Here, an in‐depth study is advanced using two more isomeric systems. Surprisingly, the results do not constantly support the reported relationship between the melting point and glass formation among isomeric molecules. Instead, molecules with enhanced glass formability are featured by the properties of low melting entropy without exception. Comprehensive studies of isomeric molecules find that the low melting entropy is roughly accompanied by the low melting point, explaining the apparent link between melting point and glass formation. Progressively, the viscosity measurements of the isomers uncover a strong dependence of the melting viscosity on melting entropy. These results emphasize the significance of the melting entropy in governing the glass formability of substances.

## Graphical abstract

To reveal the critical thermodynamic parameters determining the glass formation of substances, an in‐depth study is advanced using three isomeric systems. The results show that molecules with enhanced glass formability are featured by the properties of low melting entropy. Progressively, there is a strong dependence of the melting viscosity on melting entropy.

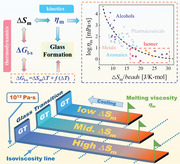

## 1 Introduction

Glassy materials are of crucial importance in industries and engineering by virtue of a number of unique properties in practical applications,^[^ ^1^, ^2^, ^3^ ^]^ but understanding glass formation has been a challenge for decades.^[^ ^4^, ^5^ ^]^ There are considerable criteria involved in the evaluation of the ability of glass formation of various substances, among which the kinetic viscosity *η* (or relaxation time *τ* and diffusion coefficient *D* ^[^ ^6^ ^]^) of melts, in particular, melting viscosity *η*_m_, is a widely accepted parameter.^[^ ^7^, ^8^, ^9^, ^10^ ^]^ It is also portrayed by two independent variables of the kinetic fragility *m* ^[^ ^11^, ^12^ ^]^ and the reduced glass temperature *T*_rg_,^[^ ^7^ ^]^ which is the ratio of the glass temperature *T_g_* and melting point *T_m_*. In contrast, there have been many thermodynamic quantities proposed to associate with glass formation, such as the Gibbs free energy difference of liquids and crystals in the undercooling regions Δ*G*,^[^ ^13^ ^]^ interface energy *σ*,^[^ ^14^, ^15^ ^]^ bond energy *E*_b_,^[^ ^16^ ^]^ mixing enthalpy Δ*H*_mix_,^[^ ^17^ ^]^ formation enthalpy Δ*H*_f_,^[^ ^18^ ^]^ mixing entropy Δ*S*_mix_,^[^ ^19^ ^]^ configurational entropy Δ*S*_conf_,^[^ ^20^ ^]^ vibrational entropy Δ*S*_vib_,^[^ ^21^ ^]^ and mismatch entropy *S*_mis_.^[^ ^22^ ^]^ Yet, these criteria are mainly applicable for evaluating the glass formation in specific alloys, and the application to more materials is challenging. Hence, a consensus remains to be clarified to pinpoint a fundamental thermodynamic parameter capable of evaluating the glass formation and guiding the composition design of glassy materials.

To seek such a fundamental thermodynamic property in governing the glass formation of substances, a simple but effective strategy is to focus on isomeric molecules because the difference in intermolecular interactions is trivial, which assists in presenting the smeared influence of other variables on the glass formation. Angell pioneered a study for the first time to understand the glass transition of three xylene isomers, proposing the low *T*_m_ principle for the glass formation in molecular isomers.^[^ ^23^ ^]^ This principle holds that the lattice of substances with high glass‐forming ability (GFA) usually has poor crystal packing manifested by the low lattice energy *E*_latt_, leading to low *T*_m_. Scrutinizing the thermodynamic data of the three xylene isomers found that *m*‐xylene (*m*‐X) with the lowest *T*_m_ (219.6 K) has the best GFA, while *T*_m_ of *o*‐xylene (*o*‐X) and *p*‐xylene (*p*‐X) is higher (247.8 K for *o*‐X and 286.4 K for *p*‐X),^[^ ^24^ ^]^ accompanied by the successively decreased glass formability. It is obvious that a relationship between GFA and *T*_m_ appears in the isomeric systems. As expected, this argument of the importance of low *T_m_* for glass formation can find more support from some criteria, for instance, the empirical rules of *T*_g_/*T*_m_^[^ ^7^ ^]^ and *T*_b_/*T*_m_,^[^ ^25^ ^]^ where *T*_b_ is the boiling point. Also, the combined criterion of *η*_m_/*T*_m_^2[^ ^26^ ^]^ in evaluating the glass formation of oxide glasses is proposed. Intuitively, lowering *T*_m_ or *T*_l_ (liquidus temperature) is advantageous to the glass formation.

Similarly, for multicomponent glassy materials such as metallic alloys, the glass formation and composition design are mainly guided using phase diagrams by pining the composition of the lowest liquidus temperature, particularly the eutectic points.^[^ ^27^, ^28^ ^]^ The temperature‐oriented composition design strategy has been prevalent for finding practically new glassy materials. However, a question as to whether the temperature‐oriented strategy is the most fundamental needs to be clarified, because no specific correlation between *T*_l_ and GFA can be verified if one considers all types of glass‐forming substances.^[^ ^12^ ^]^ Theoretically, *T*_l_ in phase diagrams is determined by the balance of the Gibbs free energies of liquid and crystal phases. Alternatively, a close inspection reveals the importance of melting entropy Δ*S*_m_ and intermolecular interaction in determining the slope of *T*_l_ in a eutectic phase diagram.^[^ ^29^ ^]^

**Figure 1:**
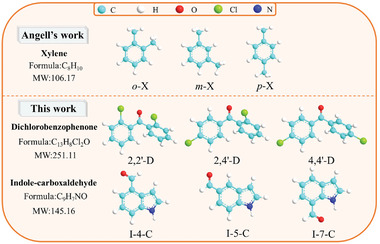
Molecular structures of experimental systems. The upper side is the system selected by Angell,^[^ ^23^ ^]^ namely, *o*‐xylene (*o*‐X), *m*‐xylene (*m*‐X), and *p‐*xylene (*p*‐X), and the lower side is the systems selected in this work, namely, 2,2′‐dichlorobenzophenone (2,2′‐D), 2,4′‐dichlorobenzophenone (2,4′‐D), 4,4′‐dichlorobenzophenone (4,4′‐D), indole‐4‐carboxaldehyde (I‐4‐C), indole‐5‐carboxaldehyde (I‐5‐C), and indole‐7‐carboxaldehyde (I‐7‐C).

In this work, we re‐checked the relation between *T*_m_ and glass formation in isomeric molecules by exploring more systems, as shown in **Figure** **1**. As the melting thermodynamics and melting viscosity are determined, the results do not show the advantage of low *T*_m_ for the glass formation. In contrast, the importance of melting entropy Δ*S*_m_ is revealed to connect closely to the glass formation. In particular, we found a strong dependence of Δ*S*_m_ on the liquid viscosity determined at *T_m_*. In‐depth analyses emphasize that Δ*S*_m_ can serve as an indicator to understand the glass formation and guide the composition design.

## 2 Results

**Figure 2:**
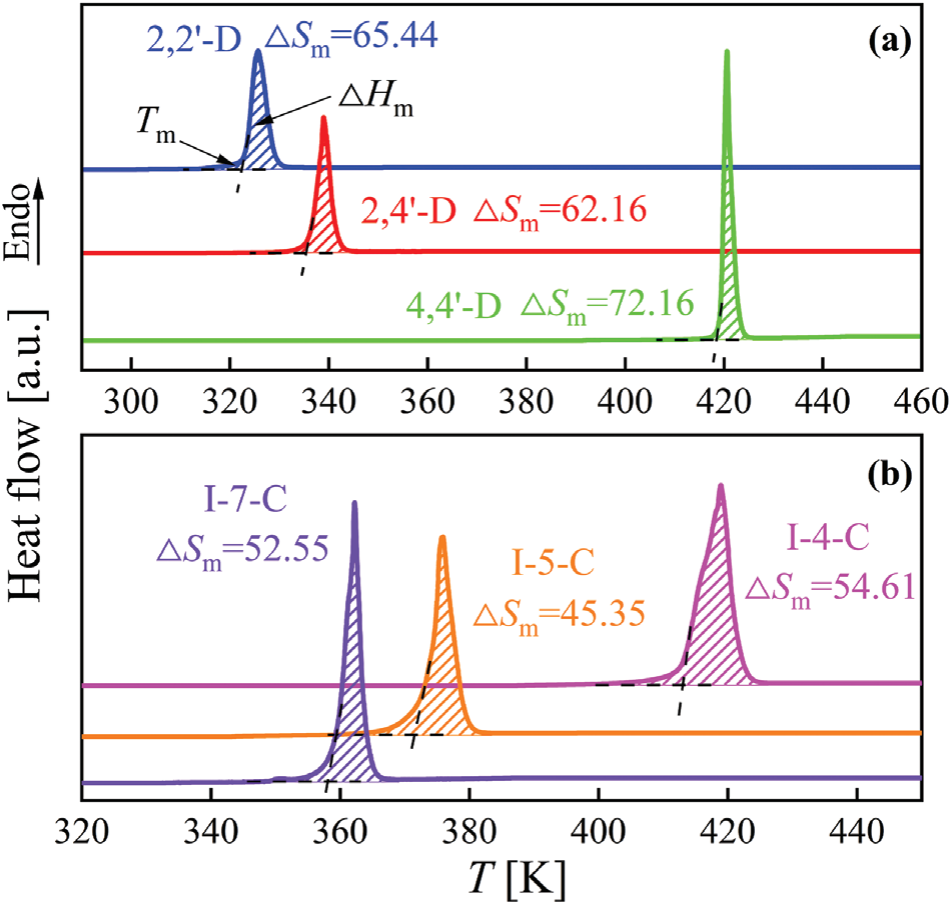
Melting DSC traces measured at the heating rate of 10 K min^−1^ of a) dichlorobenzophenone isomers and b) indole‐carboxaldehyde isomers. *T*_m_ denotes the melting point, Δ*H*_m_ is the melting enthalpy defined by the integral area of the shade regions, and Δ*S*_m_ is the melting entropy calculated by the ratio of Δ*H*_m_/*T*_m_.

**Figure** **2** shows the calorimetric melting traces of the two sets of isomers. *T*_m_ is recorded using the onset temperature of the melting traces. The melting enthalpy Δ*H*_m_ is obtained by integrating the melting peak area, and the melting entropy Δ*S*_m_ is quantified by the equation of Δ*S*_m_ = Δ*H*_m_/*T*_m_.^[^ ^30^ ^]^ The thermodynamic values are consistent with the reported ones in literature.^[^ ^31^, ^32^ ^]^ All thermodynamic characteristic quantities determined by experiments are listed in **Table** **1**.

**Table 1:** Thermodynamic, kinetic, and structural data of three sets of isomers including melting points *T*_m_, melting enthalpy Δ*H*_m_, melting entropy Δ*S*_m_, melting viscosity *η*_m_, beads, critical cooling rate *R*_c_, liquid density *ρ* at *T*_m_, and the glass‐forming ability (GFA) ranking of isomers

Substance	*T*_m_ [K]	Δ*H*_m_ [kJ mol^−1^]	Δ*S*_m_ [J mol^−1^ K^−1^]	*T*_g_ [K]	*η*_m_ [mPa s]	Beads	*ρ* [g cm^−3^]	*R*_c_ [K min^−1^]	*R*_c_^calc^ [K min^−1^]	GFA ranking
*o*‐X	247.8^a)^	13.04^a)^	52.61^a)^	126.5^b)^	1.74	3	0.84	/	27.77	2^b)^
*m*‐X	219.6^a)^	11.44^a)^	51.36^a)^	125^b)^	1.95	3	0.80	/	18.53	1^b)^
*p*‐X	286.4^a)^	16.93^a)^	59.14^a)^	/	0.71	3	0.85	/	91.98	3^b)^
2,2′‐D	323.48	22.35	65.44	215.33	3.57	3	1.20	80	13.93	2
2,4′‐D	337.28	22.11	62.16	225.56	5.75	3	1.21	10	9.48	1
4,4′‐D	420.01	30.56	72.16	/	1.89	3	1.23	>120	45.47	3
I‐4‐C	413.22	24.35	54.61	/	2.89	2	1.17	>120	47.36	3
I‐5‐C	370.43	18.85	45.35	241.24	5.21	2	1.07	20	19.31	1
I‐7‐C	359.59	19.91	52.55	215.50	2.60	2	1.15	80	39.18	2

^a)^ Reference [24]

^b)^ Reference [43].

**Figure 3:**
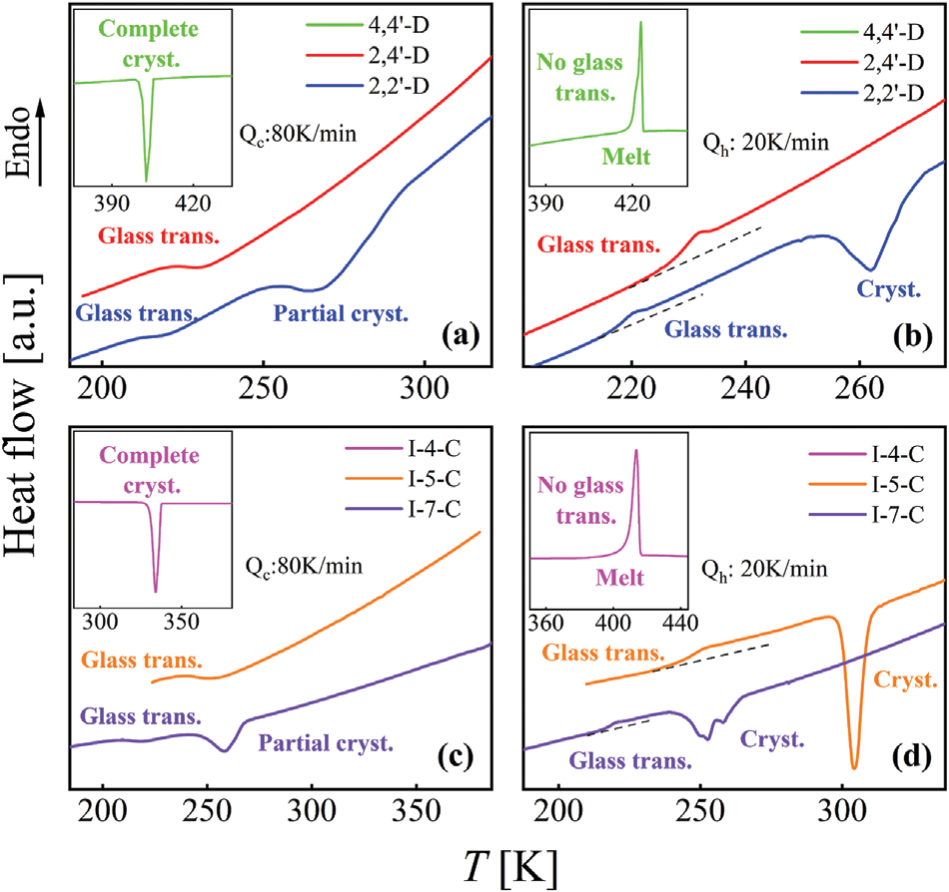
Thermograms of cooling and subsequent heating measurements of two sets of isomers. a,b) For dichlorobenzophenone, c,d) for indole‐carboxaldehyde.

**Figure** **3** shows the DSC cooling and subsequent heating curves of the two isomeric systems to monitor the glass transition behaviors. For dichlorobenzophenone isomers, vitrification occurs in both 2,2′‐dichlorobenzophenone (2,2′‐D) and 2,4′‐dichlorobenzophenone (2,4′‐D) at the highest accessible cooling rate of the calorimeter of ≈120 K min^−1^, but 4,4′‐dichlorobenzophenone (4,4′‐D) crystallized totally, indicating the worst GFA with the critical cooling rate *R*_c_ higher than 120 K min^−1^. To distinguish the GFA of 2,2′‐D and 2,4′‐D, a slower cooling rate of 80 K min^−1^ was applied while keeping the same reheating rate at 20 K min^−1^. As seen in Figure 3a, 2,2′‐D underwent partial crystallization, while the 2,4′‐D remained completely vitrified. During reheating, the glass transition of 2,2′‐D and 2,4′‐D is detected at 215.33 and 225.56 K, as shown in Figure 3b, while the 4,4′‐D directly melt at its *T*_m_. In subsequent testing of 2,4′‐D, it was found to form a complete glass at a slower cooling rate of 10 K min^−1^. Thus, *R*_c_ of 2,2′‐D and 2,4′‐D are determined to be ≈80 and lower than 10 K min^−1^, respectively. Similar experiments were performed on the indole‐carboxaldehyde isomers. The results show that indole‐5‐carboxaldehyde (I‐5‐C) and indole‐7‐carboxaldehyde (I‐7‐C) can form glasses at the highest accessible cooling rate, but the indole‐4‐carboxaldehyde (I‐4‐C) isomer is completely crystallized. Then a melt‐quenching experiment with a cooling rate of 80 K min^−1^ was applied on I‐5‐C and I‐7‐C, presented in Figure 3c. It is found that I‐7‐C is partially crystallized, while the I‐5‐C is entirely vitrified, showing the highest GFA. Figure 3d shows *T*_g_ of I‐5‐C (241.24 K) and I‐7‐C (215.50 K), meanwhile I‐4‐C only detects the melting signal. Subsequent experiments show that *R*_c_ of I‐5‐C is about 20 K min^−1^.

**Figure 4:**
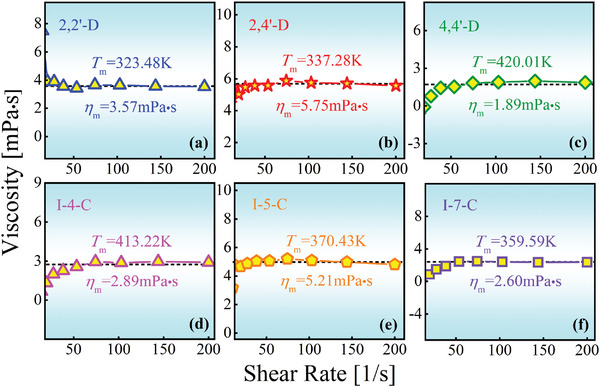
Melting viscosities *η*_m_ of dichlorobenzophenone and indole‐carboxaldehyde for a) 2,2′‐dichlorobenzophenone (2,2′‐D), b) 2,4′‐dichlorobenzophenone (2,4′‐D), c) 4,4′‐dichlorobenzophenone (4,4′‐D), d) indole‐4‐carboxaldehyde (I‐4‐C), e) indole‐5‐carboxaldehyde (I‐5‐C), f) indole‐7‐carboxaldehyde (I‐7‐C).

**Figure 5:**
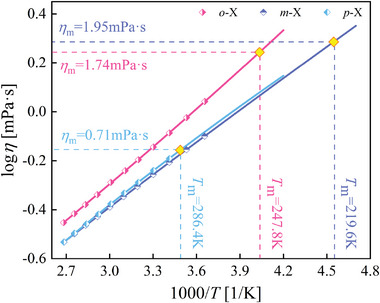
Activation plots of glass‐forming xylene isomers with the fit of viscosity data to the Vogel–Fultcher–Tammann equation. Melting viscosities *η*_m_ were calculated by extrapolating the curves to melting points *T*_m_ of xylene isomers. Viscosity data are cited from elsewhere.^[^ ^35^ ^]^

**Figure** **4** shows the viscosity results of the molecular melts measured at *T*_m_ as a function of shear rate. The viscosity of all samples tends to reach constants when the shear rate exceeds 50 s^−1^, indicating the nature of Newtonian fluids,^[^ ^33^, ^34^ ^]^ and giving the viscosity values *η*_m_ in the stable stage. Among the two sets of isomers, 2,4′‐D (5.75 mPa s) and I‐5‐C (5.21 mPa s) have the highest *η*_m_ values accompanied by their best GFA in each isomeric family. For comparison, *η*_m_ of three xylenes was obtained based on the reported results^[^ ^35^ ^]^ guided by the Vogel–Fulcher–Tammann (VFT) equation, log*η* = *A* + *B*/(*T* − *T*_0_),^[^ ^30^ ^]^ where *A*, *B* and *T*_0_ are constants. The fittings are shown in **Figure** **5**, giving *η*_m_ of xylene isomers to be 1.74, 1.95, and 0.71 mPa s for *o*‐X, *m*‐X, and *p*‐X, respectively. It can be immediately seen that *m*‐X with the highest GFA has the largest *η*_m_ value.

## 3 Discussion

Table 1 summarizes the thermodynamic and kinetic data of the isomers. To determine the accurate *R*_c_ values, which express the GFA of glass formers most directly, a calculation is introduced by referring to the experimental results. The calculated critical cooling rates *R*_c_^calc^ of three sets of isomers can be reached from the melting viscosity in terms of the quantitative relationship proposed by Sarjeant and Roy,^[^ ^36^ ^]^

(1) $$R_{c}^{\;\mathrm{calc}}=\frac{10^{-5.70}\cdot k_{B}\cdot T_{m}^{2}}{v\cdot \eta_{m}}$$

where *k*_B_ is the Boltzmann constant, *v* is the molar volume calculated from molecular weight and density. In order to gain the precise *v* values, liquid densities of the isomers at melting points are used. For isomeric xylene, the density values can be reached by using the density at room temperature together with density–temperature relations (−8.59 × 10^−4^ K^−1^ for *o*‐X, −8.81 × 10^−4^ K^−1^ for *m*‐X, and −8.89 × 10^−4^ K^−1^ for *p*‐X).^[^ ^35^ ^]^ For the other two families of molecular isomers, the calculated results are in the same order of magnitude as the experimental results when using the liquid densities at *T*_m_s. Earlier studies have also examined and confirmed the reliability of this formula.^[^ ^37^, ^38^, ^39^, ^40^, ^41^, ^42^ ^]^ Inspecting experimental and calculated *R*_c_ together with *η*_m_ of the isomers shown in Table 1, it is immediately seen that low *T*_m_ does not always suggest the enhanced GFA for each isomeric family studied here. This is somehow a surprise and arises a question as to whether there is a thermodynamic parameter, which can predict the glass formation more accurately.

In Angell′s studies,^[^ ^43^ ^]^ the interpretation of the highest GFA of the meta isomer emphasizes the lowest lattice energy *E*_latt_ with the lowest *T*_m_.^[^ ^23^ ^]^ *E*_latt_ depends on intermolecular interaction force and molecular packing fashion, indicating that low *E*_latt_ is accompanied by relatively loose packing structures with low *T*_m_. Decreased *T*_m_ is critical in enhancing *η*_m_, which hinders the migration and rearrangement of molecules or atoms during nucleation and growth and inhibits the precipitation of crystal phases from the liquid, consequently favoring glass formation.^[^ ^6^ ^]^ Thus, the low *T*_m_ effect on glass formation is an indication of the unique crystal phases and the slow liquid kinetics.

Yet, the correlation between *T*_m_ and the glass formation becomes challenging when more materials are involved for comparison. Simple examples are metal alloys, for which there is no direct correlation between low *T*_l_ and GFA.^[^ ^12^, ^44^ ^]^ Moreover, holding the consensus of the kinetic criterion of the glass formation that *η*_m_ is the dominant factor in determining glass formation,^[^ ^7^, ^8^, ^9^, ^10^ ^]^ the relation of both *T*_l_ and the Gibbs free energy of liquid and solid phases together with their difference of Δ*G*_l‐s_ to *η*_m_ remains vague.

Earlier studies of small‐molecule glass formers found that *T*_g_ is positively correlated with the boiling point *T*_b_, which can largely express the intermolecular interaction.^[^ ^45^ ^]^ This implies that *T*_g_ can also serve as an indicator of interaction force, as emphasized in earlier studies.^[^ ^46^ ^]^ By contrast, *T*_m_ depends on intermolecular interaction forces and the crystal packing fashion. Based on Angell's argument of the advantage of low *T*_m_ for the glass formation together with the interpretation of the relationship of *T*_m_ and unique crystalline structures of isomeric glass formers, one can expect a large proportion of low‐frequency vibration modes due to the low lattice energy and the low crystalline packing efficiency. This would suggest higher vibrational entropy in such crystals.^[^ ^47^ ^]^ When assuming that the entropy of different liquid alloys has no pronounced difference due to the disordered states at individual melting points, the low melting entropies Δ*S*_m_ would be expected for the isomers with high GFA. With the thermodynamic data of each group of isomeric molecules listed in Table 1, it is evident that Δ*S*_m_ shows a closer correlation with GFA. A similar conclusion can also be reached if one inspects the thermodynamic results of glass‐forming metal alloys by *Busch*’s group, showing that low Δ*S*_m_ favors glass formation.^[^ ^48^, ^49^ ^]^ It is also seen in Table 1 that isomeric molecules with lower melting entropies basically hold higher glass transition temperatures. Similar observations have been reported in earlier studies of poly (*ε*‐caprolactone) and nylon‐6, which hold the virtually identical conformations,^[^ ^50^, ^51^ ^]^ and show the negative relation between Δ*S*_m_ and *T*_g_. Our recent studies of various glass formers have also implied that there are other thermodynamic parameters than *T*_l_ or *T*_m_ that could determine the glass formation.^[^ ^12^, ^29^, ^30^ ^]^

**Figure 6:**
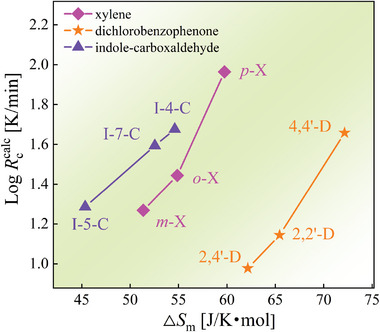
Dependence of calculated glass‐forming critical cooling rate *R*_c_^calc^ on melting entropy Δ*S*_m_ for three types of isomeric molecules of xylenes (X), dichlorobenzophenones (D), and indole‐carboxaldehydes (I‐C).

**Figure** **6** presents a quantitative connection between Δ*S*_m_ and GFA expressed by *R*_c_^calc^ for the three sets of isomers. The correlations are quite acceptable, confirming the significance of low Δ*S*_m_ in mediating the glass formation of materials, compared with several reported thermodynamic ones.^[^ ^17^, ^18^, ^19^, ^20^, ^21^, ^22^, ^23^ ^]^ This is not an exception since there is increasing evidence reported in recent studies that Δ*S*_m_ has a series of correlations with glass formation related properties. For instance, studies of eutectic systems with negative heat of mixing Δ*H*_mix_ show a deficit in Δ*S*_m_,^[^ ^52^ ^]^ where negative Δ*H*_mix_ is generally regarded as an essential condition for glass‐forming systems.^[^ ^53^ ^]^ Moreover, based on the metallic compounds of low Δ*S*_m_, glassy materials have been successfully designed and prepared such as a semiconductor glass of Ga_2_Te_3_‐SnTe^[^ ^54^ ^]^ and bulk metallic glasses of (Zr_40_Ti_40_Ni_20_)_100‐x_Be_x_.^[^ ^55^ ^]^ Theoretically, the Gibbs free energy difference Δ*G*_l‐s_ between liquid and solid phases is associated with glass formation due to its nature of the crystallization driving force. Δ*G*_l‐s_ can be expressed in terms of^[^ ^13^ ^]^ Δ*G*_l − s_ = Δ*S*_m_Δ*T* + *f*(Δ*T*), where Δ*T* is the degree of supercooling relative to *T*_m_, and *f* (Δ*T*) is the higher‐order function. Lower Δ*G*_l‐s_ is required to suppress the crystallization, corresponding directly to a lower Δ*S*_m_.^[^ ^56^ ^]^ Similarly, the studies of the crystal growth in melts found that the system with low Δ*S*_m_ has a lower growth rate,^[^ ^57^ ^]^ favoring to suppress crystallization and, thus, enhancing the glass formation. The significance of Δ*S*_m_ on the glass formation mentioned above allows for the conclusion that Δ*S*_m_ might be able to serve as a fundamental thermodynamic property and to make contribution to the glass formation, compared with a number of reported thermodynamic ones.^[^ ^17^, ^18^, ^19^, ^20^, ^21^, ^22^, ^23^ ^]^ Earlier studies have revealed that the melting entropy Δ*S*_m_ of materials is a crucial thermodynamic quantity involved in many aspects of crystals and liquids such as crystal growth,^[^ ^57^ ^]^ crystal structure,^[^ ^58^ ^]^ and clustering in liquids.^[^ ^59^ ^]^

Yet, one more key question needs to be addressed regarding how glass formation thermodynamics and kinetics work jointly in governing the glass formation. Given the fact that glass‐forming compositions can be guided by phase diagrams in the vicinity of the composition of deep eutectics, it can be inferred that thermodynamics can independently guide the glass formation, since phase diagrams can be interpreted by the thermodynamic Gibbs free energy of liquids and crystals. Moreover, a close inspection of the liquidus in phase diagrams, it can be easily found that the melting entropy is the fundamental property to control the slope of the liquidus, and a low melting entropy usually arises a high slope, favoring the occurrence of deep eutectics.^[^ ^29^ ^]^ Alternatively, one can understand the glass formation from the perspective of kinetics. Commonly, it is accepted that kinetic melting viscosity *η*_m_ is a dominant quantity in governing the glass formation.^[^ ^6^, ^60^ ^]^ This indicates that kinetics can basically determine the glass formation, as evidenced by Equation (1), suggesting the dominant effect of the kinetics on the glass formation. Such a paradox of the glass‐forming thermodynamics and kinetics might find its solution by seeking certain correlation between the two aspects. Albeit the viscosity of glass formers has not been well explained,^[^ ^61^ ^]^ there are indeed some clues presenting links between viscosity and thermodynamic quantities. For instance, Adam–Gibbs established the dependence of melt viscosity on configurational entropy Δ*S*_conf_,^[^ ^20^ ^]^ which is a faction of the liquid‐crystal entropy difference, which becomes Δ*S*_m_ at *T*_m_. Also, Greet and Magill found that melt viscosity can be interpreted by melting entropy Δ*S*_m_.^[^ ^62^ ^]^ Nevertheless, establishing a direct correlation between Δ*S*_m_ and *η*_m_ in different types of materials keeps a tremendous challenge, because melting entropy Δ*S*_m_ depends numerically on molecular structure, indicating that Δ*S*_m_ of a molecule with more atoms would be higher. This leads to a consequence that Δ*S*_m_ of molecules with different atom number is not comparable with regard to the glass formation. This differs much from the kinetic quantities such as melting viscosity *η*_m_, which is numerically independent of the atom number of molecules.

**Figure 7:**
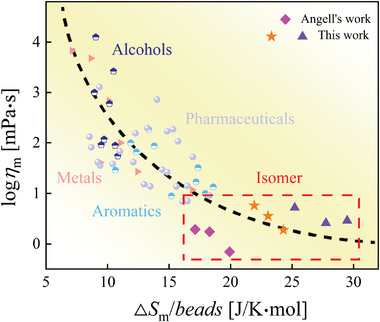
Correlation between melting viscosity *η*_m_ and melting entropy Δ*S*_m_ for various glass‐forming liquids covering molecular and metallic systems. The dashed line is a guide for the eye.

To explore the correlation between Δ*S*_m_ and *η*_m_ for different types of glass formers, a concept of normalized Δ*S*_m_ by beads is proposed and applied successfully in our recent studies.^[^ ^30^ ^]^ The beads concept was initially proposed by Wunderlich^[^ ^63^ ^]^ to describe intramolecular degrees of freedom and quantified by the number of rotatable units, having an equivalent physical implication to the number of excitable degrees of freedom per molecule.^[^ ^64^, ^65^ ^]^ Recent reports have also indicated that the beads value of molecules may be identified by measuring OH bond orientation polarization using infrared spectra.^[^ ^66^ ^]^ **Figure** **7** presents the relation of normalized Δ*S*_m_ and *η*_m_ for the isomeric systems studied in this work together with xylene isomers, as represented in our previous study.^[^ ^30^ ^]^ The link between *η*_m_ and Δ*S*_m_ is observed for glass formers such as metals,^[^ ^5^, ^67^, ^68^, ^69^, ^70^, ^71^, ^72^, ^73^, ^74^, ^75^ ^]^ aromatic,^[^ ^76^, ^77^, ^78^, ^79^, ^80^, ^81^ ^]^ alcohols,^[^ ^47^, ^82^, ^83^ ^]^ and pharmaceuticals.^[^ ^30^, ^83^, ^84^, ^85^ ^]^ Since isomers have the same beads values, the normalization does not change the relation between Δ*S*_m_ and *η*_m_. This holds true in each isomeric system, showing lower Δ*S*_m_ corresponds to higher *η*_m_ and stronger GFA.

In addition to the studies of the glass formation thermodynamics and kinetics, structural consideration for the glass formation has been also extensively studied from the perspective of the similarity and difference of liquid and crystal structures. For example, recent studies have proposed that crystal nuclei are not born randomly but induced in regions of high crystal‐like bond‐orientational order in a supercooled liquid, and the manipulation of the crystallization kinetics was achieved by controlling the degree of liquid preordering.^[^ ^86^, ^87^ ^]^ It seems that the appearance of a crystal‐like structure in the liquid is beneficial to nucleation. Note that the similarity or the difference in the structures of the liquid and its crystals can be reflected by the melting thermodynamics, in particular, the melting entropy.^[^ ^58^, ^59^ ^]^

Finally, the advantages of using isomers to clarify the fundamental thermodynamic quantity in mediating the glass formation should be highlighted, in particular, for understanding the contributions made by enthalpy and entropy. Among molecular isomers, the difference in enthalpy is markedly reduced, and, thus, the entropy effect on the glass formation can be well resolved. In contrast, for the conventional comparison of the GFA among various materials with a large difference in melting points, the entropy effect was usually smeared because of the large difference in enthalpy, and thus, the independent contributions made by enthalpy and entropy are hardly achieved.

## 4 Conclusion

Isomeric molecules were selected to identify the key thermodynamic quantity in determining the glass formation. With the reported results, the critical issue of the glass formation thermodynamics is clarified, indicating that the melting entropy is more correlated with the glass formation than the melting point of a system, and the GFA of the systems with low melting entropy is stronger. Experimental evidence also demonstrates that for the glass‐forming systems, the melting entropy has a close connection to melting viscosity, indicating that the melting entropy of a system not only serves as the driving force of crystallization, but has an impact on the glass formation by regulating the kinetic viscosity. Thus, the melting entropy of a system is a fundamental thermodynamic parameter in determining glass formation.

## 5 Experimental Section

The samples studied here are two sets of isomers of dichlorobenzophenone and indole‐carboxaldehyde, including 2,2′‐dichlorobenzophenone (2,2′‐D, Alfa, 98%), 2,4′‐dichlorobenzophenone (2,4′‐D, TCI, 99.0%), 4,4′‐dichlorobenzophenone (4,4′‐D, TCI, 99.0%), indole‐4‐carboxaldehyde (I‐4‐C, TCI, 98.0%), indole‐5‐carboxaldehyde (I‐5‐C, TCI, 98.0%), and indole‐7‐carboxaldehyde (I‐7‐C, Sigma–Aldrich, 97.0%). The corresponding molecular structures are shown in Figure 1, and all the samples are used without further purification.

A Perkin–Elmer differential scanning calorimeter (Diamond DSC) was used to measure the glass transition and melting behaviors. The DSC equipment was calibrated with indium and cyclohexane. Powder samples were sealed in aluminum pans and melted at a heating rate of 10 K min^−1^ from *T*_m_ −30 K to *T*_m_ +30 K to probe melting processes. Varied quenching rates were performed for samples to determine the crystallization tendency of the molecules from their melt states. The glassy states were attained by quenching the molten samples to low temperatures at the highest cooling rate (≈120 K min^−1^),^[^ ^52^ ^]^ and then reheating was performed to get the glass transition signals at a fixed heating rate of 20 K min^−1^. Based on the accessible cooling rates for the glass formation in the isomers, the ranking of GFA can be reached, as listed in Table 1. Smaller number corresponds to enhanced GFA. To obtain the liquid densities, Archimedes principle was applied by monitoring the weight change of a tungsten rod immersed into the liquid isomers heated to 30 K above *T*_m_ to ensure complete melting. And the density of the molecular liquids was calculated by,^[^ ^88^ ^]^

(2) $$\rho =\frac{\rho_{0}\left(m_{0}-m_{1}\right)}{m_{1}}$$

where *ρ*_0_ is the density of the tungsten rod, *m*_0_ is the actual weight of the tungsten rod, and *m*_1_ is the weight of the tungsten rod immersed into the liquids.

The kinetic viscosity was determined using the MCR‐302 rheometer from Anton Paar equipped with the pp25 parallel plates. The samples were placed between two parallel plates spaced by a distance of 4 mm and heated to 10 K above their *T*_m_. The molten samples were isothermally maintained for 5 min to guarantee complete melting. Then the temperature was decreased and stabilized to the corresponding *T*_m_ of each sample, and a shear rates of 0.01–200 s^−1^ were applied to the samples.

## Conflict of Interest

The authors declare no conflict of interest.

## Data Availability

The data that support the findings of this study are available from the corresponding author upon reasonable request.
